# Promoting implementation of “Good and Best Practices” through Health Promotion and Prevention Registries

**DOI:** 10.1093/eurpub/ckac129.444

**Published:** 2022-10-25

**Authors:** C Rossmann, D van Dale, S Rados-Krnel, M Kylanen, K Lewtak, M Grasso, C Tortone, P Ragazzoni, L Costa, A Maassen

**Affiliations:** 1 Department for Cross Sectional Research, Federal Centre for Health Education Germany, Cologne, Germany; 2 Department of Health and Society, RIVM, Bilthoven, Netherlands; 3 Development of Health, National Institute of Public Health, Ljubljana, Slovenia; 4 Finnish Institute of Health and Welfare, Helsinki, Finland; 5 Department of Health Promotion and Chronic Diseases, National Institute of Public Health – National Institute of Hygiene, Warsaw, Poland; 6 DoRS - Health Promotion Regional Documentatio, Piedmonte Region, Italy; 7 Department of Health Promotion and NCD's, National Institute of Health Doutor Ricardo J, Lisbon, Portugal; 8 EuroHealthNet European Partnership for Health, Brussel, Belgium

## Abstract

Health promotion and disease prevention programme registries can play an important role in increasing transparency of “good/ best Practices” and promoting their implementation. In the EU, there are different approaches to how registries seek to support the implementation of practices. However, there is limited knowledge of the extent to which there are informative differences or overlaps in the mechanisms chosen for accreditation, capacity building and implementation. This presentation focuses on six national registries in the EU (Finland, Germany, Italy, Netherlands, Poland, Slovenia) and the European Public Health Best Practice Portal. Information was obtained through a working group on Good/ Best Practice Portals, established in 2019. Information on the process of adding good/best practices to the registry and on measures that promote implementation was gathered and evaluated through a descriptive case comparison. The comparison suggests that implementation can be promoted through different approaches for capacity building measures and incentives for implementation. The latter included funding resources, titling/awarding of practice and professional feedback in only a few registries. Registries may be useful not only for transparency of good and best Practices but also for implementation through capacity building and several incentives. The information provided in this presentation may be informative in guiding development of similar resources elsewhere and a starting point for discussion on how to support best the implementation of good/ best practices.

